# Biological characteristics of SARS-CoV-2 resistant populations by integrated gut microbiota sequencing, metabolomics, and proteomics: a cohort comparison study

**DOI:** 10.3389/fmed.2025.1593007

**Published:** 2025-07-07

**Authors:** Huachong Xu, Haoxuan Li, Junhao Xu, Yaoxin Chen, Li Deng, Xiaoyin Chen, Yinji Xu

**Affiliations:** ^1^School of Traditional Chinese Medicine, Jinan University, Guangzhou, Guangdong, China; ^2^Zhongshan Hospital of Traditional Chinese Medicine, Zhongshan, Guangdong, China; ^3^The Second Clinical College, Guangzhou University of Chinese Medicine, Guangzhou, China; ^4^Department of Pulmonary and Critical Care Medicine, Zhuhai Hospital of Integrated Traditional Chinese and Western Medicine, Zhuhai, Guangdong, China; ^5^Guangdong Provincial Hospital of Chinese Medicine, Guangzhou, Guangdong, China

**Keywords:** SARS-CoV-2, COVID-19, resistant population, multiomics, phosphatidylinositols, gut microbiota, metabolomics, proteomics

## Abstract

**Objective:**

Most research reports on COVID-19 infections have focused on the correlation between the severity of the disease symptoms and immune deficits, while the mechanisms affecting the susceptibility to SARS-CoV-2 remain largely unknown. The study aimed to comprehensively analyze the differences in immunity, gut microbiota, metabolism, and proteomics between the SARS-CoV-2 resistant population and the susceptible population.

**Methods and results:**

In this cohort comparison study, participants were rigorously selected based on inclusion and exclusion criteria in a continuous enrollment manner using combined questionnaires and clinical data, ultimately including 25 SARS-CoV-2 resistant volunteers versus 16 SARS-CoV-2 infected patients. The clinical information of the participants was recorded in detail, and fecal and blood samples were collected in a standardized manner for subsequent multi-omics analysis, including gut microbiota sequencing, metabolomics, and proteomics. This study has preliminarily elucidated the characteristics of the gut microbiota, serum metabolites, and serum proteins in the SARS-CoV-2 resistant population. It exhibits a unique metabolic signature characterized by elevated levels of serum phosphatidylinositol and the abundance of Prevotella, which may serve as a potential predictive biomarker for resistance to SARS-CoV-2.

**Conclusion:**

Given the crucial role of phosphatidylinositol in cell membrane architecture and viral infectivity, this study provides a promising entry point for further research into the pathogenesis and prevention strategies of COVID-19.

## 1 Introduction

SARS-CoV-2 is capable of causing novel coronavirus infections (COVID-19), a highly contagious and fatal infectious disease (1–3), which has had profound impacts on health and economies worldwide (4, 5). Most research reports on COVID-19 infections have focused on the correlation between the severity of the disease symptoms and immune deficits (6), while the mechanisms affecting the susceptibility to SARS-CoV-2 remain largely unknown. It is particularly noteworthy that, despite the widespread pandemic, there remains a subset of the population that has consistently not been infected, including those who work in high-risk environments but have consistently tested negative for nucleic acids or antigens (7, 8). These individuals are commonly considered to have a natural resistance to SARS-CoV-2 and are referred to as the SARS-CoV-2 resistant population (7, 8).

Why have these unvaccinated individuals who are repeatedly exposed to high-risk contact with SARS-CoV-2 not shown evidence of COVID-19 during the pandemic? Among possible explanations, host factors have been proven to drive susceptibility to SARS-CoV-2 and the severity of the COVID-19 disease (9). Human susceptibility to SARS-CoV-2 is influenced by various factors, including gender, age, exposure environment, genetic background, number of vaccinations, gut microbiota, and immune response (10–12), and there is significant variation in the individual immune system’s ability to detect and respond to SARS-CoV-2. Therefore, a comprehensive understanding of the heterogeneity in immunity, metabolism, gut microbiota, etc., between COVID-19 patients and the SARS-CoV-2 resistant population is crucial for the prevention, treatment, and prognosis of the disease. Multi-omics can provide a three-dimensional analysis of each aspect of the biological process, understanding biological phenomena and mechanisms from a systems-level perspective (6). For instance, proteomic studies mapping SARS-CoV-2-human protein interactions identified druggable host targets (13), while metabolomics profiling revealed a six-lipid panel with near-perfect diagnostic accuracy (14). Integrated transcriptomic, proteomic, and metabolomic analyses further uncovered immune dysregulation patterns: severe COVID-19 correlates with neutrophil hyperactivity and IFN-I signaling, whereas mild cases show robust T-cell responses (15).

To screen for individual differences in resistance or susceptibility to SARS-CoV-2, this study included a clinical, prospective observational study of the SARS-CoV-2 resistant population and the infected population. On the basis of testing basic clinical biochemical and immunological indicators, this study employed various omics technologies, including gut microbiota sequencing, metabolomics, and proteomics, to analyze serum, feces, and other samples from both the SARS-CoV-2 resistant and infected populations. We aim to comprehensively analyze the differences in immunity, gut microbiota, metabolism, and proteomics between the SARS-CoV-2 resistant population and susceptible population, thereby identifying the factors most closely related to susceptibility or resistance to SARS-CoV-2, to provide a more complete idea for revealing the pattern of COVID-19 infection occurrence.

## 2 Materials and methods

### 2.1 Trial design

In this dual-cohort comparison study, the SARS-CoV-2 resistant population (group B) included in this study mainly comes from individuals with a higher risk of clinical exposure to SARS-CoV-2 since 1 December 2022, and have tested negative for the SARS-CoV-2 RNA or antigen tests. They mainly include medical staff, interns, or university laboratory personnel from the Guangdong Provincial Hospital. The SARS-CoV-2 infected patients (group A) included in the study are mainly from the outpatient or inpatient departments of the Guangdong Provincial Hospital, matching the age and gender of the SARS-CoV-2 resistant population and having similar SARS-CoV-2 exposure characteristics. They are faced with equal exposure risks and adhered uniformly to protective measures such as isolation gowns, N-95 respirators and safety goggles ensuring comparable levels of protection and exposure across individuals. Basic personal information of the two groups of individuals were collected and blood, feces, saliva, etc. Samples were collected and stored for subsequent blood routine tests, SARS-CoV-2 antibody testing, as well as 16S gut/saliva microbiota, metabolomics, proteomics sequencing analysis. The study was approved by the Ethics Committee of Guangdong Provincial Hospital of Traditional Chinese Medicine (ZE2023-009-01) and approved by Chinese Clinical Trial Registry (ChiCTR2400094191, Primary Registry of WHO International Clinical Trials Registry Platform).

### 2.2 Diagnostic criteria and classification basis

Referring to the “Diagnosis and Treatment Plan for SARS-CoV-2 Coronavirus Pneumonia (10th edition)” issued by the Chinese National Health Commission: relevant clinical manifestations of SARS-CoV-2 infection and positive results on pathogenic or serological testing. Primary clinical manifestations include dry throat, sore throat, cough, and fever, which is mostly low or moderate, sometimes high, lasting no more than 3 days.

Some patients may also experience muscle aches, loss or reduction of smell and taste, nasal congestion, runny nose, diarrhea, conjunctivitis, etc. Pathogenic and serological testing results: (1) positive COVID-19 RNA test for SARS-CoV-2; (2) positive antigen test for SARS-CoV-2; (3) positive virus isolation and culture for SARS-CoV-2; and (4) recovery phase SARS-CoV-2 specific IgG antibody levels increase by four times or more compared to the acute phase.

### 2.3 Inclusion and exclusion criteria

#### 2.3.1 Inclusion criteria for the SARS-CoV-2 resistant population group

① Age ≥ 18 years, no gender preference; ② individuals with a higher risk of clinical exposure to SARS-CoV-2 since 1 December 2022, and have tested negative for the SARS-CoV-2 RNA or antigen tests based on epidemiological history, clinical manifestations, laboratory tests, etc.; ③ no related respiratory infection symptoms such as fever, cough, sore throat in the past 3 weeks; and ④ participants must sign a written informed consent form to participate in the study.

#### 2.3.2 Inclusion criteria for the SARS-CoV-2 infected patients group

① Age ≥ 18 years, no gender preference; ② according to the “Diagnosis and Treatment Plan for SARS-CoV-2 (10th edition)” issued by the Chinese National Health Commission, diagnosed as patients with SARS-CoV-2 infection based on epidemiological history, clinical manifestations, and laboratory tests; ③ onset within 1 week, clinically classified as mild or moderate; and ④ participants must sign a written informed consent form to participate in the study.

#### 2.3.3 Exclusion criteria

① Individuals with chronic respiratory diseases such as chronic obstructive pulmonary disease, pulmonary interstitial fibrosis, bronchial asthma, obstructive sleep apnea-hypopnea syndrome, or a history of lung surgery; ② individuals with cardiovascular, liver, kidney, hematological, and neurological diseases, malignant tumors, or immune dysfunction (including immunosuppressive drug use or HIV infection causing immune deficiency); ③ individuals with common cold or influenza, acute pharyngitis, tonsillitis, rhinitis, sinusitis, other types of upper respiratory tract infections, pneumonia, bronchitis, or other lower respiratory tract infections triggered by influenza or common cold; ④ individuals with severe gastrointestinal diseases, experiencing severe diarrhea (more than 3 watery stools and lasting more than 3 days) and constipation (less than 2 bowel movements per week with difficulty in passing stool) in the past 3 weeks; ⑤ overweight individuals [body mass index (BMI) > 28.0 kg/m^2^], pregnant or lactating women; ⑥ non-compliant individuals who cannot cooperate with clinical observation and sample collection; and ⑦ other factors leading to clinical dropout.

### 2.4 Experimental grouping

Recruitment of the SARS-CoV-2 resistant population and SARS-CoV-2 infected patients was conducted through publicity in the outpatient and inpatient departments of the Guangdong Provincial Hospital and some communities in Guangzhou. Using a combination of questionnaires and clinical data, participants were strictly selected based on inclusion and exclusion criteria in a continuous enrollment manner, with 25 SARS-CoV-2 resistant volunteers and 16 SARS-CoV-2 infected patients included in the study respectively.

### 2.5 Basic information and sample collection

#### 2.5.1 General information collection

Collect the gender, age, height, weight of the enrolled individuals, calculate the BMI; record the course of illness (for SARS-CoV-2 infected patients)/history of SARS-CoV-2 exposure (for the SARS-CoV-2 resistant population), medical history, SARS-CoV-2 vaccination history, smoking/drinking habits, allergies, respiratory symptoms, and medication information.

#### 2.5.2 Clinical laboratory testing indicators

Peripheral venous blood samples were collected from fasting participants for testing serum total IgE levels, two types of SARS-CoV-2 antibodies, five immunological items, hypersensitive C-reactive protein, complete blood count (CBC), T lymphocyte subset (percentage) detection, and carcinoembryonic antigen. Specimens were destructed immediately after testing.

#### 2.5.3 Collection of fecal microbiota samples

Before collecting fecal samples, participants were advised to eat high-water content foods such as vegetables and fruits and drink plenty of water to facilitate bowel movement. They were provided with fecal sample collection tubes containing preservation solution, and after successful collection within 3 days, the samples were returned/sent back to the hospital for numbering and freezing at −80°C. Sufficient dry ice was used for shipment and subsequent metagenomic analysis. Note: Fecal samples were destructed immediately after completion of metagenomic testing.

#### 2.5.4 Serum sample collection

Fasting peripheral venous blood was drawn from participants and collected in vacuum blood collection tubes without anticoagulants (red cap). The blood was allowed to clot and form layers by standing at room temperature for 1 h. Using a medical low-speed centrifuge at 3,000 rpm for 10 min at room temperature, the blood was separated, and the serum was transferred to clean centrifuge tubes. Finally, a high-speed centrifuge at 12,000 rpm for 10 min at 4°C was used to transfer the serum to centrifuge tubes or cryogenic tubes, approximately 0.5 ml per tube. To ensure integrity, multiple tubes were filled as much as possible and stored in the refrigerator at a freezing temperature of −80°C. Adequate dry ice was used for shipment to ensure sample stability for subsequent metabolomics and proteomics analysis.

### 2.6 Clinical indicator testing organization and methods

Inflammatory and immune-related indicators of the two groups of patients were tested. The detection of SARS-CoV-2 IgG antibody (chemiluminescence method) and SARS-CoV-2 IgM antibody (chemiluminescence method) were purchased from Bioscience (Chongqing) Biotechnology Co., Ltd., with medical device registration number 20203400183 for IgG and 20203400182 for IgM. C-reactive protein was tested using the immunoturbidimetric method and the reagent kit was purchased from Roche Diagnostics Products (Shanghai) Co., Ltd., with medical device registration number 20212400184.

### 2.7 Microbiome sequencing, metabolomics, and proteomics detection methods and analysis methods

#### 2.7.1 Detection methods and analysis methods of fecal microbiome sequencing

This study revealed the microbial composition of different samples through 16S rDNA sequencing and complex data analysis. DNA extraction was performed using the CTAB method, followed by PCR amplification with primer design specific to each sample. PCR products were validated and purified through gel electrophoresis, then sequenced using a high-throughput sequencing platform. Sequencing results were transferred to the Illumina NovaSeq platform. Paired-end reads were assigned to samples based on their unique barcodes, and the barcodes and primer sequences were trimmed. Flash was used to merge paired-end reads. The original reads were quality-filtered using fqtrim (v0.94) to obtain high-quality clean tags. Vsearch software (v2.3.4) was used to filter chimeric sequences. After deduplication with DADA2, feature tables and sequences were obtained. Alpha and beta diversity were calculated by normalizing to the same random sequence. Relative abundance of features for each sample was normalized using the SILVA (release 138) classifier. Alpha diversity was calculated using QIIME2, including Chao1, observed species, Good’s coverage, Shannon, Simpson indices, etc. These indices for our samples were calculated and plotted using R packages. Beta diversity was calculated using QIIME2, and graphical representations were created using R packages. Sequence alignment was performed using Blast, and representative sequences were annotated using the SILVA database. Other charts were created using the R language (v3.5.2). Differential abundance of bacterial taxa was analyzed using the multivariate statistical model MaAsLin2. Bacterial taxa with an abundance > 0.05% in at least one sample, an adjusted *P*-value < 0.05 [Benjamini–Hochberg false discovery rate (FDR) correction], and a FC > 1.5 were considered statistically significant.

#### 2.7.2 Detection methods and analysis methods of metabolomics sequencing

The method for extracting metabolites from serum samples involved adding each sample to a mixture of 500 μl acetonitrile-methanol-water (2:2:1, v/v/v) with isotopically labeled internal standard mix. After vortex mixing for 30 s, the samples were sonicated at 35 Hz for 4 min and then ultrasonicated in an ice-water bath for 5 min, repeated three times. Samples were then stored at −40°C for 1 h, centrifuged at 12,000 × *g* for 15 min at 4°C, and the supernatant (400 μl) was transferred to EP tubes and vacuum-dried, then dissolved in 50% acetonitrile. Subsequently, 75 μl of the supernatant was used for liquid chromatography-tandem mass spectrometry (LC-MS/MS) analysis. An equal amount of all supernatants was taken as quality control (QC) samples. For serum samples, 50 μl of serum was added to a mixture of 200 μl methanol-acetonitrile (1:1, v/v) with isotopically labeled internal standard mix, followed by the same procedure. Finally, the raw metabolomic data was processed, including peak detection, extraction, alignment, and integration, followed by multivariate analysis such as principal component analysis (PCA) and orthogonal partial least squares-discriminant analysis (OPLS-DA). Differential metabolites were identified through variable importance in the projection (VIP) and statistical analysis, followed by hierarchical clustering analysis (HCA) and heatmap construction, as well as pathway enrichment analysis. Given the untargeted nature of our LC-MS platform, reported metabolite differences reflect relative abundance based on normalized peak intensities rather than absolute concentrations. Metabolite quantification was conducted using XCMS software to extract signal intensities across samples, followed by QC and preprocessing with metaX software: low-quality peaks (missing in >50% of QC samples or >80% of actual samples) were removed, missing values were imputed via the *K*-nearest neighbors (KNN) method, and data normalization was sequentially performed using probabilistic quotient normalization (PQN) and QC-robust spline batch correction (QC-RSC). Post-normalization, ions with a coefficient of variation (CV) >30% in QC samples were excluded due to high experimental variability. For differential metabolite analysis, a hybrid approach combining univariate statistics [fold-change (FC) and Student’s *t*-test with Benjamini–Hochberg (BH)-adjusted *q*-values] and multivariate analysis [partial least squares discriminant analysis (PLS-DA)-derived VIP scores] was applied. Differential ions were defined by simultaneous thresholds: |log2(FC)| ≥ 0.58 (equivalent to FC ≥ 1.5 or ≤1/1.5), *P*-value ≤ 0.05, and VIP ≥ 1.

#### 2.7.3 Detection methods and analysis methods of proteomics

In this experiment, 4D-DIA technology was used for proteomics analysis. Serum samples underwent protein separation, followed by analysis using a nanoElute chromatography system and timsTOF Pro mass spectrometer. During the experiment, proteins were first separated by SDS-PAGE electrophoresis, enzymatically digested using the FASP method, and peptide segments were separated by High pH RP fractionation. Protein data was obtained through DDA mass spectrometry library construction and DIA mass spectrometry analysis. Protein data was functionally annotated and pathway annotated using Gene Ontology (GO) and Kyoto Encyclopedia of Genes and Genomes (KEGG) databases, followed by enrichment analysis. Bioinformatics analyses such as subcellular localization analysis, domain annotation and enrichment analysis, clustering analysis, and interaction network analysis were conducted. Differential protein analysis was conducted by first selecting the sample pairs for comparison. The FC for each protein was calculated as the ratio of the mean quantitative values across all biological replicates in the compared sample pairs. To assess statistical significance, a Student’s *t*-test was performed on the relative quantitative values of each protein between the two sample groups, generating a *P*-value as the significance metric (default threshold: *P*-value < 0.05). In this study, upregulated proteins were defined as those with FC > 1.5 and *P*-value < 0.05, while downregulated proteins were defined as those with FC < 0.67 and *P*-value < 0.05. Volcano plots were generated by plotting the logarithmic FC [log2(FC)] against the absolute logarithmic *P*-value [−log10 (*P*-value)]. HCA was performed on the relative abundance of differentially expressed proteins across samples, visualized via a heatmap to observe upregulation and downregulation patterns. Each row was normalized using *Z*-scores, calculated as (observed value − row mean) / row standard deviation.

### 2.8 Statistical methods description

For data collection and entry, a database was established using Epidata V.3.1 software. All data was entered by two individuals. After logical checking, validation, cleaning, and blind review, the database was locked, and analysis was conducted using SPSS V.22.0, while statistical graphs were created using Graph Pad Prism 9 or R software. Continuous data was expressed as “mean ± standard deviation” or median/interquartile range. For comparisons between two groups, one-way analysis of variance (ANOVA) was used when the variance was homogeneous, and the Kruskal–Wallis test was used when the variance was not homogeneous. Categorical data was represented as composition ratios or rates, and intergroup comparisons were made using Fisher’s exact test (2 × C), while rank-sum test (Mann–Whitney method) was used for ordinal data. Statistical methods for microbiome, metabolomics, and proteomics can be found in section “2.7 Microbiome sequencing, metabolomics, and proteomics detection methods and analysis methods.”

## 3 Results

### 3.1 General characteristics of the study population

**FIGURE 1:**
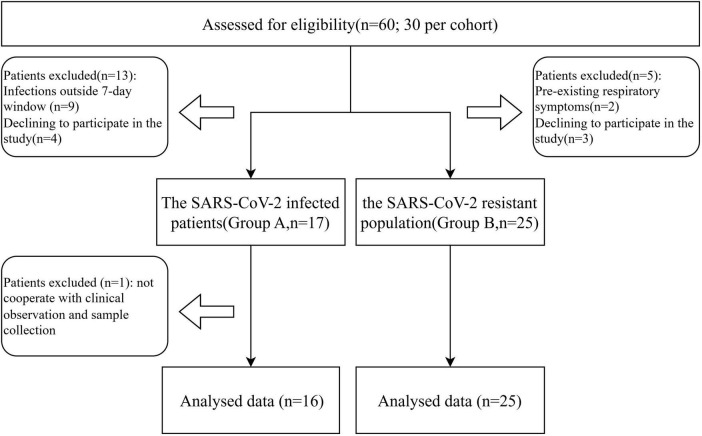
Participant flow diagram.

Throughout the duration of the trial, which spanned from 12 January 2023, to 11 February 2023, a cohort of 60 individuals participated in the study. A total of 19 subjects were excluded and one further exclusion occurred in group A due to not cooperate with clinical observation and sample collection (Figure 1). Within this cohort, 16 subjects were identified as patients infected with SARS-CoV-2 (group A), whereas the remaining 25 were classified as the SARS-CoV-2 resistant population (group B). All participants underwent the complete suite of examinations. Patient demographics and clinical characteristics at the time of screening are detailed in Table 1. At the study’s inception, no significant disparities were observed between the two groups with respect to age, gender, BMI, alcohol consumption history, smoking history, allergic history, medical history, or the number of vaccinations received. It was observed that individuals within the SARS-CoV-2 resistant population group exhibited a preference for a balanced dietary structure between animal and plant foods.

**TABLE 1:** Baseline Characteristics of the Study Cohorts.

Characteristic		Group B (*n* = 25)	Group A (*n* = 16)	*P*-value
Age (years), Mean		33.04	27.63	0.15
Sex	Male (*n*)	10	8	0.53
	Female (*n*)	15	8	
BMI (kg/m^2^), Mean		22.61	22.35	0.84
Alcohol consumption (*n*)		1	0	/
History of cigarette smoking (*n*)		0	0	/
Allergy history (*n*)		5	7	0.10
Past medical history (*n*)		3	2	0.96
Number of SARS-CoV-2 vaccines, Mean		2.76	2.81	0.25
Dietary patterns	Meat-based with vegetarian supplements (*n*)	4	8	0.05*
	Vegetarian-based with meat supplements (*n*)	2	0	
	Balanced structure of animal and plant foods (*n*)	19	8	

BMI, body mass index. “*” indicates *P* < 0.05 for comparison between two groups.

### 3.2 Clinical immune indexes and complete blood count analysis between the SARS-CoV-2 resistance group and the infection group

**FIGURE 2:**
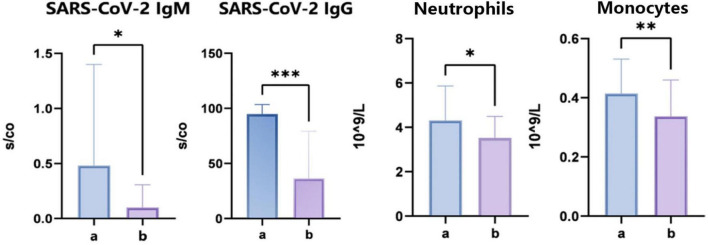
Clinical immune indexes and complete blood count analysis. “*” indicates *P* < 0.05 for comparison between two groups. “**” indicates *P* < 0.01 for comparison between two groups. “***” indicates *P* < 0.001 for comparison between two groups.

Upon admission, serum specimens were collected from each participant for subsequent analysis. We conducted an evaluation of 12 clinical parameters in groups A and B, which included total IgE concentrations, five immunological markers, high-sensitivity C-reactive protein (hs-CRP), T lymphocyte subset percentages, and carcinoembryonic antigen levels falling within normal physiological ranges. However, we observed significant differences in the levels of SARS-CoV-2 IgM (*P* = 0.0126) and IgG (*P* = 0.001), suggesting disparate infection statuses among the groups. The SARS-CoV-2-resistant group showed lower antibody expression. Additionally, during the CBC analysis, there were significant variations noted in the counts of neutrophils (*P* = 0.043) and monocytes (*P* = 0.001) (Figure 2). The above data indicate that there are some differences in immune profiles between the COVID-19 resistance group and the infection group, which may be related to the infection mechanism.

### 3.3 Differences in gut microbiota between the SARS-CoV-2 resistance group and the infection group

**FIGURE 3:**
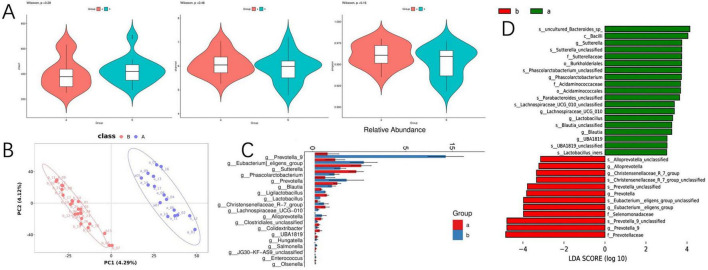
Differences in gut microbiota between the SARS-CoV-2 resistance group and the infection group. **(A)** Violin diagram of alpha diversity; **(B)** partial least squares discriminant analysis of gut microbiota; **(C)** the result chart of differential bacterial genera screening at the genus level between the COVID-19 infection group and the COVID-19 resistant group; **(D)** linear discriminant analysis effect size analysis; microbial features were classified based on both statistical significance and biological effect size.

To assess the variations in gut microbiome diversity between the two cohorts, we initially quantified the alpha diversity of fecal specimens utilizing metrics such as Chao1, Shannon, and Simpson indices. Our analysis indicated no significant disparities in alpha diversity between the groups as presented in Figure 3A. Further, a PLS-DA was employed to dissect the community structure of the gut microbiota. The cohorts manifested distinct segregation along the principal coordinate axis 1 (PC1), which underscores compositional distinctions in gut microbiota as detailed in Figure 3B. This finding implies that despite a uniform spectrum of microbial varieties across the cohorts, their proportional abundances are substantially divergent. Differential fecal microbiota compositions were evident between the groups, as denoted in Figure 3C. For instance, relative to group A, there was an escalation in the prevalence of *Prevotella_9, Eubacterium eligens, Prevotella, Ligilactobacillus, Christensenellaceae_R-7_group, Alloprevotella, Salmonella, Enterococcus, and Olsenella in Group B. Inversely, the quantities of Sutterella, Phascolarctobacterium, Blautia, Lactobacillus, Lachnospiraceae_UCG-010, Clostridiales, Colidextribacter, UBA1819, Hungatella*, and *JG30-KF-AS9* unclassified were diminished in group B.

In continuation, linear discriminant analysis (LDA) allied with effect size estimates (LEfSe) was harnessed to ascertain the significance of the observed microbial community shifts and relative enhancements across groups A and B. At the genus level, LEfSe analysis unveiled a pronounced decrement in the genera *Sutterella, Phascolarctobacterium, Lachnospiraceae_UCG_010, Lactobacillus, Blautia*, and *UBA1819*, concomitant with an increment in *Alloprevotella, Christensenellaceae_R-7_group, Prevotella, Eubacterium_eligens_group*, and *Prevotella_9* in group B as cataloged in Figure 3D. In the aforementioned microbial communities, we observed associations between specific taxa (e.g., Prevotella and Enterococcus) and phosphatidylinositol (PI) metabolism, consistent with prior reports linking these microorganisms to lipid signaling pathways (16, 17).

### 3.4 Differentially abundant metabolites related to SARS-CoV-2 resistance group

**FIGURE 4:**
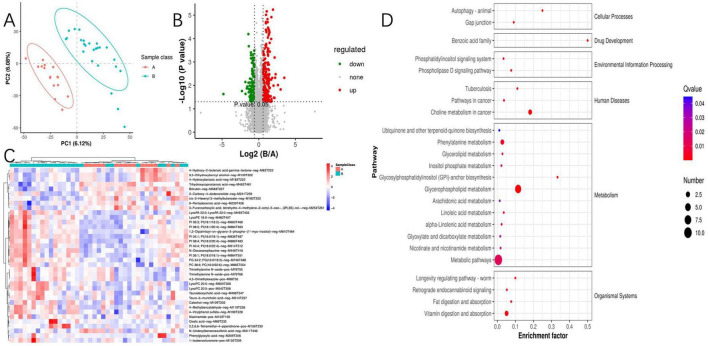
Between-group comparative analysis of serum metabolites. **(A)** Partial least squares discriminant analysis of metabolites; **(B)** volcano plot for differential metabolites; **(C)** metabolomics cluster analysis plot; **(D)** Kyoto Encyclopedia of Genes and Genomes enrichment pathway of metabolites.

Considering the intricate relationship between the gut microbiome and host metabolic processes, we embarked on untargeted metabolomics investigations of serum samples. The deployment of OPLS-DA unveiled distinct metabolic profiles between group A and group B, signifying substantial modifications in the serum metabolite composition (Figure 4A). Through stringent selection criteria of a VIP greater than 1.5 and a *P*-value less than 0.05, we pinpointed 37 unique metabolites. Notably, group B exhibited an upregulation of 27 metabolites, inclusive of 15 glycerophospholipid-associated metabolites, whereas a downregulation of 10 metabolites was observed (Figures 4B, C). In pursuit of a deeper understanding of the differential metabolites’ metabolic pathways and functions, we engaged in a thorough analysis utilizing the Human Metabolome Database (HMDB) and the KEGG online databases. The analysis indicated that the differential metabolites were primarily concentrated in pathways related to glycerophospholipid metabolism, choline metabolism in cancer, phenylalanine metabolism, and vitamin metabolism (Figure 4D). A further probe into glycerophospholipid metabolism revealed six metabolites associated with PI metabolism, all upregulated in group B. This observed association between PI metabolism and susceptibility phenotypes suggests a potential mechanistic link worthy of further experimental validation. To augment our insights, we performed a comprehensive joint analysis of fecal microbiota and serum metabolomics.

### 3.5 Associations between the metabolome, clinical parameters, and gut microbiome

**FIGURE 5:**
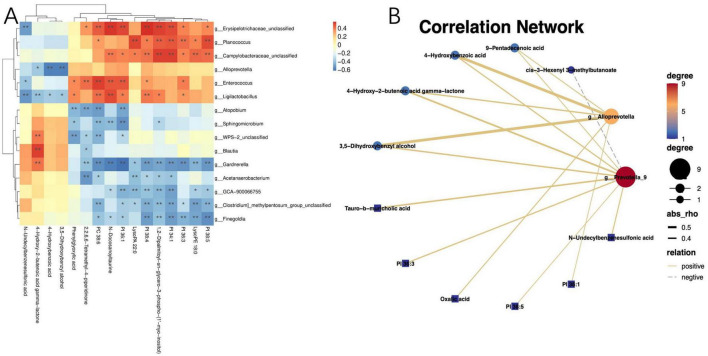
Correlation between the gut microbiota and metabolites. **(A)** Heatmap of joint analysis of metabolomics and gut microbiota; **(B)** diagram of differential metabolites and differential microbiota network regulation. “*” indicates *P* < 0.05 for comparison between two groups. “**” indicates *P* < 0.01 for comparison between two groups.

Given the profound physiological impact of the gut microbiota on its host, which often facilitated through a complex host-microbe metabolic axis, our study further explored the relationships between the prevalence of specific bacterial genera and certain metabolites within the gut microbiome. We discovered a correlation between the presence of potentially pathogenic Enterococci, Prevotella, etc. and an increase in serum metabolites, including PI 38:5, PI 36:3, PI 34:1, 1,2-dipalmitoyl-sn-glycero-3-phospho-(1′-myo-inositol), PI 38:4, and PI 36:1 (Figures 5A, B). Utilizing Spearman’s rank correlation analysis and selecting data with a rho value below −0.4 or above 0.4 and a *P*-value less than 0.05, we identified that metabolites such as PI 36:3, PI 34:1, PI 38:4, and PI 38:5 are significantly negatively correlated with COVID-19 IgG antibody levels (Table 2). These findings robustly endorse the hypothesis that alterations in the mechanisms of COVID-19 susceptibility are intricately regulated by a dynamic host-microbe metabolic axis, exhibiting significant associations with PIs (Figures 5A, B).

**TABLE 2:** Correlation between metabolites and COVID-19 IgG antibody levels.

Target	Metabolites	Rho	*P*-value	Relation
COVID-19 IgG antibody levels	PI 36:3; PI (18:1/18:2)	−0.52138	0.000473	Negative
	PI 34:1; PI (16:0/18:1)	−0.50845	0.000688	Negative
	PI 38:4; PI (18:0/20:4)	−0.4795	0.001513	Negative
	PI 38:5; PI (18:1/20:4)	−0.43116	0.004887	Negative

### 3.6 Joint analysis of proteomics and metabolomics

**FIGURE 6:**
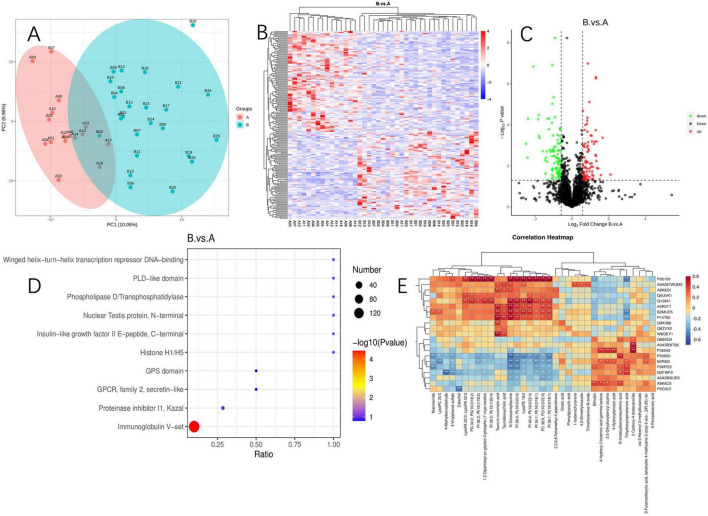
Proteomics and its correlation between metabolites. **(A)** Principal component analysis of proteomics; **(B)** proteomics cluster analysis plot; **(C)** volcano plot of differential protein; **(D)** differential protein enrichment pathway plot; **(E)** heatmap of joint analysis of differential metabolites and differential proteins. “*” indicates *P* < 0.05 for comparison between two groups. “**” indicates *P* < 0.01 for comparison between two groups. Protein identifiers and their functional descriptions are provided in Supplementary Table 1.

To further elucidate the mechanisms underlying the changes in PI metabolism, we conducted a proteomic analysis of serum samples followed by PCA, which revealed significant protein differences between the two groups (Figures 6A, B). Among the quantifiable 713 proteins, a total of 177 proteins exhibited significant differential expression between group A and group B, with 82 proteins upregulated and 95 proteins downregulated (FC > 1.5 or <0.67 and *P*-value of <0.05) (Figure 6C). Differential expression proteins were analyzed through the GO database, identifying the liver histone H1e protein, which is enriched in pathways including nucleosome, nucleus, and nucleosome assembly. Histone H1 and its variant, H1e, are classified as linker histones, playing a crucial role in binding DNA between nucleosomes to facilitate the compaction and stabilization of chromatin into higher-order structures. This process is vital for the efficient packaging of DNA within the cell nucleus and exerts significant regulatory influence on gene expression patterns. The designation “e” in H1e specifies a particular subtype or isoform of the H1 histone, characterized by unique expression profiles and functional roles across varying cell types and physiological states.

We conducted KEGG enrichment analysis to identify the metabolic pathways associated with differentially expressed proteins. Among the upregulated proteins, there was a significant enrichment in pathways such as the PI3K-Akt signaling pathway, B cell receptor signaling pathway, Fc epsilon RI signaling pathway, NF-κB signaling pathway, natural killer cell-mediated cytotoxicity, and the phospholipase D signaling pathway. Additionally, we identified the 5-3 exonuclease PLD4, which is associated with PI metabolism.

Enrichment analysis of protein domains among differentially expressed proteins can predict the functions of proteins with unknown roles. The enrichment of protein domains is illustrated in Figure 6D, showing that the differentially expressed proteins predominantly contain domains such as “Immunoglobulin V-set,” “Proteinase inhibitor I1 Kazal,” “GPCR, family 2, secretin-like,” “GPS domain,” and “Histone H1/H5.” The V-set domain is functionally diverse, regulating chromosomal structure, participating in DNA replication and repair; it modulates gene expression and protein methylation and plays a pivotal role during viral invasion. Similarly, through an integrated analysis of proteomics and metabolomics, we discovered that Histone H1e(A3ROT7) is significantly associated with PI metabolism (Figure 6E).

## 4 Discussion

This study aimed to explore the intestinal flora, serum metabolites, and related serum proteomic changes that can explain the susceptibility difference of COVID-19, and try to explore the related biological mechanism through the joint analysis of multiomics. We have preliminarily elucidated the characteristics of the gut microbiota, serum metabolites, and serum proteins in the SARS-CoV-2-resistant population. This group exhibits a unique metabolic signature characterized by elevated levels of serum PI, which may serve as a potential predictive biomarker for resistance to SARS-CoV-2.

Individual susceptibility to SARS-CoV-2 varies greatly, with these differences partly stemming from genetic factors. For instance, the ACE2 protein plays a crucial role in the infection process of SARS-CoV-2, with its level of expression directly affecting an individual’s sensitivity to the virus. However, genetic factors alone cannot fully explain the susceptibility to COVID-19. Studies have noted that the rate of infection among children living in the same household is lower than that of their parents (18), and there is a significant increase in the incidence rate among individuals over the age of 75 (19). In numerous families where almost all members were infected, there was often a single individual who remained uninfected (20), suggesting that some individuals who are highly exposed to SARS-CoV-2 may possess resistance to the infection. Since the disease was first described in December 2019, there has been substantial progress in understanding the pathophysiology of life-threatening COVID-19. However, research focusing on virus resistance has been much less common than research on susceptibility, leaving the biological basis of innate resistance to SARS-CoV-2 largely unexplored.

With the advancement of multi-omics technologies, researchers are gaining an increasingly sophisticated understanding of the gut microbiome, serum metabolites, and their significant roles in regulating immune responses and the development of infectious diseases. The borderline statistical significance of dietary pattern differences observed in the cohort, potentially attributable to the limited sample size and reduced statistical power, necessitates cautious interpretation of dietary confounding effects. While these findings might suggest a subtle influence of diet on gut microbiome composition, the microbial signatures differentiating SARS-CoV-2-resistant and infected individuals demonstrate biological plausibility independent of dietary factors. Prior evidence independently associates gut microbiota with antiviral immunity, reinforcing the hypothesis that host-pathogen interactions, rather than dietary variability alone, underpin the observed microbial patterns (21, 22). Nevertheless, the interplay between diet, microbiome dynamics, and immune responses remains an important consideration. To resolve this ambiguity, future large-scale studies with standardized dietary monitoring are critical to disentangle the contributions of SARS-CoV-2 resistance, dietary habits and their synergistic effects. In the analysis of the gut microbiome, we first compared the species differences between the SARS-CoV-2 resistant population and the infected population. No statistical difference was found in alpha diversity between the two groups. Therefore, we further employed PLS-DA and found a clear distinction between the two groups, indicating a statistical difference in the gut microbiome structure between the SARS-CoV-2 resistant and infected groups. Currently, there is no consensus on the relationship between the alpha diversity and beta diversity of the gut microbiome and the resistance or susceptibility to COVID-19. Many studies suggest significant differences in both alpha and beta diversity between healthy controls and COVID-19 patients (23, 24), but some studies show no statistical difference in species diversity between the gut microbiome of COVID-19 patients and that of healthy individuals (25). Further analysis of the key differential genera revealed that the abundances of three genus: *Prevotella_9*, *Prevotella*, and *Alloprevotella* were increased in the SARS-CoV-2 resistant group compared to the infected group, with *Prevotella_9* showing a significant change in proportion. Further LEfSe analysis also indicated that Prevotella was predominantly enriched in the SARS-CoV-2 resistant group. *Prevotella* is a Gram-negative, non-motile rod-shaped bacteria that exist as single cells and can grow in anaerobic environments. This bacterium is considered associated with a healthy vegetarian diet and plays a beneficial role in the human body. This aligns with the baseline preference of the SARS-CoV-2 resistant population for a balanced diet rather than an imbalanced one. There are also reports that Prevotella is associated with the pathogenesis of COVID-19. A study analyzed the Prevotella proteins secreted by respiratory Prevotella and found multiple interactions with the NF-κB pathway (26). Overexpressed Prevotella proteins can promote viral infection, leading to an increase in the clinical severity of COVID-19, suggesting that Prevotella might play a role in the outbreak of COVID-19 (26). Another study showed that patients with COVID-19 pneumonia had a greater fecal abundance of Prevotella compared to those with mild infections (27). Further research found that, compared to mild and asymptomatic cases, moderate COVID-19 patients had a depletion of the genus Prevotella in their gut microbiome (28). These findings suggest that the Prevotella genus may play an important role during COVID-19 infection. Given the current insufficient understanding of the pathological mechanisms by which the gut microbiome affects COVID-19 resistance and the limited evidence linking Prevotella with susceptibility to the virus, this study suggests that the role of Prevotella in resistance and susceptibility to COVID-19 requires further investigation.

While the diversity of the gut microbiota has been identified, relying solely on these differences to fully explain the resistance and susceptibility to COVID-19 is insufficient. The gut microbiota influences the human body in various ways, including the modulation of serum metabolites. Gut microorganisms can break down dietary fibers and other components, producing a variety of metabolites such as short-chain fatty acids (SCFAs), amino acids, and vitamins (29). These metabolites are absorbed through digestion and circulated throughout the body via the bloodstream, where they regulate the function of immune cells and trigger anti-inflammatory or pro-inflammatory responses, helping to maintain the balance of the immune system (29). To explore whether the dysbiosis of the gut microbiota in COVID-19 patients leads to metabolic disorder that subsequently affects resistance and susceptibility to COVID-19, this study conducted metabolomic analysis on the serum of two groups of individuals. Through the analysis of differential metabolites, 37 distinct metabolites were identified between the group with resistance to SARS-CoV-2 and the group with infections. Of these, 28 metabolites were upregulated and 9 were downregulated; among the upregulated differential metabolites, 7 were PIs.

Phosphatidylinositol is a crucial component of cell membranes, involved in cellular signaling and regulation of cell functions. Through the action of specific enzymes, PI can be converted into various phosphorylated forms, allowing it to rapidly respond to external signals, modulate the spatial structure of membrane proteins, stabilize adjacent membrane contact sites for ion exchange and signal transduction between cells, and adjust the cytoskeleton to maintain normal cellular activities. Some studies have found statistical differences in PI levels between COVID-19 patients and healthy controls, with PI levels capable of distinguishing between the two groups and correlating with the severity of the disease (30, 31). Researchers speculate that abnormal reductions in PI may be related to the structure of the cellular endoplasmic reticulum. The endoplasmic reticulum is a complex membrane network that is ubiquitously distributed in the cytoplasm and stably connected to almost all organelles. This membrane characteristic creates a lipid gradient and corresponding metabolic pathways at specific locations, leading to more significant changes in phospholipid metabolites than in other metabolites (32). Further studies have found that (33) COVID-19 patients have higher anti-PI antibodies (0.223) compared to healthy controls (0.103), and PI is a major component of the SARS-CoV-2 envelope (34). Phosphatidylinositol could affect the binding efficiency of the virus to host cell receptors, thereby influencing viral particle endocytosis and release. This study conducted a correlation analysis between Prevotella, which showed the greatest difference between groups, and PI, the metabolite with the most significant intergroup difference, and found a clear positive correlation between Prevotella and PI in COVID-19 patients. It is also noteworthy that a significant negative correlation was found when correlating COVID-19 virus antibody IgG with PI. Therefore, this study postulates that the group resistant to SARS-CoV-2 may have higher serum levels of PI, which could also be positively correlated with the abundance of gut Prevotella. This could be one of the potential mechanisms that reduce the binding efficiency of the virus to the host cell receptors, thereby increasing resistance to SARS-CoV-2.

To further elucidate the underlying mechanisms governing alterations in PI metabolism, we engaged in a proteomic analysis of serum samples. Through an integrated approach combining proteomics with metabolomics, we discerned a significant correlation between histone H1e and PI metabolism. Histones, including H1 and its variant H1e, are classified as linker histones and play a pivotal role in binding DNA between nucleosomes to facilitate chromatin compaction and stabilization into higher-order structures. This process is vital for the efficient packaging of DNA within the nucleus and exerts a substantial regulatory effect on gene expression patterns. Subsequently, we conducted a KEGG enrichment analysis of the differential proteins and found a significant enrichment in pathways including the PI3K-Akt signaling pathway, B-cell receptor signaling pathway, Fc epsilon RI signaling pathway, NF-κB signaling pathway, natural killer cell-mediated cytotoxicity, and the phospholipase D signaling pathway, among others. Specifically, the PI signaling system, activated via PI3K, generates diacylglycerol (PIP2) and triphosphoinositide (PIP3), which in turn activates downstream signaling molecules such as protein kinase B (AKT), involved in processes encompassing cellular proliferation, survival, and differentiation. This suggests that the aforementioned pathways may be the potential mechanisms through which a cohort resistant to COVID-19 effectuates a lower susceptibility via PI signaling.

Nevertheless, our study is subject to some limitations. On one hand, the sample size is relatively small, impacted by the rapid spread of multiple outbreaks and the diminished population resistant to COVID-19. Future efforts to increase the sample size and to undertake multicenter studies will enhance the robustness of the findings. On the other hand, multi-omics approaches have revealed distinct subsets of gut microbiota, differentially abundant serum metabolites, and host proteins associated with COVID-19 resistance. While integrated analyses have uncovered potential biological pathways, the mechanistic underpinnings at the genetic level remain uncharacterized. However, these findings ultimately demonstrate correlational relationships, necessitating mechanistic studies to validate the specific pathways hypothesized through bioinformatic analyses. In future research, we intend to conduct more in-depth studies to clarify the host’s systemic adaptations involved in combating COVID-19, such as those mediated by the gut–lung axis, rather than just a single immune response. Building on previous research on COVID-19 prevention, treatment, and rehabilitation (35–38), we will further explore the potential application value of specific biomarkers of resistant populations (e.g., characteristic gut microbiota and related metabolites) in interventions across these stages.

## 5 Conclusion

In summary, this study identifies preliminary microbial and metabolic signatures associated with SARS-CoV-2 resistance in our cohort, characterized by serum PI elevation and Prevotella enrichment. While these correlations suggest testable hypotheses about host-microbe interactions in viral susceptibility, they require validation through controlled mechanistic studies—such as fecal microbiota transplantation in animal models or targeted metabolic interventions. This study provides a promising entry point for further research into the pathogenesis and prevention strategies of COVID-19. Beyond its immediate applications, the multi-omics framework adopted in this work establishes a scalable methodological pipeline that highlights host-microbe metabolic crosstalk as a critical yet underexplored axis in epidemic resilience. By integrating gut microbiota sequencing, metabolomics, and proteomics, this approach offers a methodological reference for rapidly identifying resistance biomarkers in future infectious disease crises, while advances the integration of susceptibility analysis into researchers’ methodological frameworks probably.

## Data Availability

The data presented in this study are deposited in the MetaboLights repository, accession number MTBLS12654, https://www.ebi.ac.uk/metabolights/MTBLS12654.
